# YOGA, TAI CHI, AND PILATES AND FAMILY CAREGIVERS’ SUBJECTIVE WELL-BEING: FINDINGS FROM MIDUS

**DOI:** 10.1093/geroni/igad104.2528

**Published:** 2023-12-21

**Authors:** Kallol Kumar Bhattacharyya, Yin Liu, Elizabeth Fauth

**Affiliations:** Utah State University, Logan, Utah, United States; Utah State University, Logan, Utah, United States; Utah State University, Logan, Utah, United States

## Abstract

Being an informal caregiver has been associated with stress and lower levels of subjective well-being. Exercise/movement therapy techniques of yoga, tai chi, and Pilates are physical activities that also incorporate stress reducing practices. The current study aimed to examine the association of exercise/movement therapy techniques on family caregivers’ subjective well-being assessed by life satisfaction and mindfulness. Participants were part of the Midlife in the United States (MIDUS) study (wave 2, N = 507 aged 35-86 years; Mage = 56±11, 68% women) who provided informal care in the past 12 months. We recoded the aforementioned exercise/movement therapy activities into three categories, based on the distributions in MIDUS, including regular practice (defined as participating in one or more of them “a lot” or “often”), irregular practice (participating “sometimes” and “rarely”), and no practice (“never”). Subjective well-being was measured using the 5-item global life satisfaction scale and the 9-item mindfulness scale. We considered sociodemographic factors and health as covariates. We used multivariate linear regression models to examine associations between practice and caregivers’ subjective well-being. After controlling for covariates, the findings indicated that regular practice was associated with both better mindfulness-related well-being (b = 2.26, p < .05) and better life satisfaction (b = 0.43, p < .05). Future research should examine whether there is a selection effect of caregivers with higher well-being being more likely to choose these activities, and/or if exercise/movement therapies are effective non-pharmacological interventions to improve family caregivers’ overall quality of life.

